# Augusta Academy of Medicine

**Published:** 1894-10

**Authors:** 


### *SOCIETY REPORTS.*

---

### AUGUSTA ACADEMY OF MEDICINE.

AUGUSTA, GA., August 1, 1894.

*President Jos. Eve Allen, M.D., in the Chair.*

DR. CHAS. J. MONTGOMERY read a paper on "Tetanus." (See page 452.)

The President opened the discussion by saying that he had had very little experience with traumatic tetanus, but that he had studied carefully tetanus neonatorum, and had published the results of his investigations in the April, 1894, number of the *American Journal of Obstetrics.* From his observation of over one hundred cases, he was satisfied that trismus of new-born infants was an acute infectious disease, due to the absorption of septic matter (ptomaines) from the umbilical fossa, and could be entirely prevented by strict aseptic precautions in the dressing of the cord.

He wished to call particular attention to an error in diagnosis which is often made between trismus and the epileptic convulsions of young infants. Epileptic convulsions are met with after tedious and instrumental labors, and are due to pressure upon or other injury to the brain. They come on much earlier than the convulsions of tetanus, generally within the first twelve or twenty hours after birth, while tenatus does not make its appearance until between the fifth and ninth day. These epileptiform attacks are often very severe, but the prognosis is much more favorable than in true tetanus, for as soon as the brain becomes accommodated to the pressure of the depressed bone or blood-clot the convulsions cease. The diagnostic point to be remembered is that in epileptic convulsions there is complete muscular relaxation between the attacks, while in tetanus the child never relaxes, but becomes more drawn with every spasm. He also reported a case of fatal puerperal tetanus. The patient, a white primipara, aged twenty-six years, first had uremic eclampsia; was then delivered after a long and severe instrumental labor; recovered entirely from the eclampsia and appeared to be perfectly well until the ninth day, when she was suddenly seized with tetanus and died on the eleventh day. He asked if the excited state of the nervous system consequent upon the eclampsia rendered the woman more susceptible to the tetanic poison.

DR. JAS. B. MORGAN said that he had met with tetanus more frequently in those persons who worked about horses, such as stablemen, drivers, etc., a fact that had been noted by other observers. He thought that the offal of horses offered a culture-field peculiarly favorable to the growth and development of the tetanus bacillus.

DR. W. W. BATTEY stated that he relied in the treatment of tetanus upon quinine, chloral, and chloroform. With reference to the connection of horses with tetanus, he said that in certain islands of the Malay Archipelago, where horses were unknown, the natives, when they go to war, dip their arrows and spears in soft swamp mud and allow it to dry on them. Wounds with these weapons invariably produced tetanus.

DR. J. H. CARMICHAEL said that he believed that the germs of tetanus were indigenous to certain localities, as he had recently treated two traumatic tetanus cases in the same neighberhood, that had developed within a few days of each other.

DR. DES. FORD said that he was not willing to subscribe entirely to the germ theory of tetanus, because he had seen cases develop when the wound was perfectly aseptic and every precaution of modern surgery had been observed. Tetanus was often due to the peripheral nerves being dragged upon during the process of cicatrization, or irritated by small spiculæ of bone. The lockjaw of infants could be traced to careless handling, rubbing, or pulling upon the stump of the cord. He called attention to the observations of Dr. J. Marion Sims, which showed that it was frequently caused by the depressed occipital bone pressing upon the base of the brain. Often in traumatic tetanus a cure followed the excision of the scar. He said that in all his experience as a railroad surgeon, he had never seen tetanus follow wounds received on the line of road. It sometimes happened, however, after slight injuries in the shops. The treatment should be mainly directed toward obtunding the sensibilities of the nervous system, and in opium we had an agent that best subserved this purpose. He regarded opium as the sheet anchor of the therapeutics of tetanus. In all the cases that have recovered under his care this was the drug used. He had seen cases of trismus nascentium get well under drop doses of laudanum, often repeated, and he was often astonished at the great tolerance of infants of opiates. The inestimable value of opiates in conditions of great nervous excitement had early been impressed upon him by the teachings of his father, Dr. Lewis D. Ford, and Dr. L. A. Dugas.

DR. THOS. R. WRIGHT confirmed Dr. Ford's statement as to the occurrence of tetanus after wounds that had been treated aseptically, and reported a fatal case after a breast amputation, in which the disease did not appear until the wound had entirely healed.

DR. H. H. MALONE had seen in a horse tetanus of about two weeks' duration cured by the administration of fluid extract of cocoa leaves after all other remedies had failed.

DR. W. H. DOUGHTY, JR., said that he had no practical experience with tetanus, as he could not remember having ever treated a case; from his study of the subject, however, he was certain that the disease was of microbic origin. The bacillus belonged to what is known as the anaërobic group of microbes,—that is, it was one of those germs that could not grow in presence of air. This explained the fact that tetanus usually followed punctured or small wounds, and those that healed rapidly, thus shutting out the air, large lacerated wounds, where a great deal of surface was exposed, were rarely followed by tetanus. A punctured wound that quickly closed presented exactly the conditions for the culture of tetanus germs that were found in the test tube of the bacteriologist. The spores and ptomaines formed slowly, and hence the disease appeared late, often long after the traumatism had been cured. In those cases where tetanus supervened after wounds had been treated aseptically, the sterilization had been carried only far enough to destroy the staphylococcus pyogenes aurens and the streptococcus pyogenes, the pus-producing bacteria, and had not been sufficient to kill the spores of the tetanus bacillus. It required a moist heat of over 100° C., maintained for an hour or more, to effect this result. The treatment should consist in keeping the wound aseptic by irrigation with some germicide rich in oxygen, such as peroxide of hydrogen. He did not think that hydrate of chloral and opium were the best agents for internal use. Viewed from the standpoint of its physiological action, he regarded bromide of potassium as the drug most indicated. It should be given until it produced its full physiological effect; the reason it had heretofore been of no benefit was because it was given in too small doses. The dose of a drug should not be regulated by rules laid down in books, but by its effect upon the case in hand; and hence if it required pound doses of bromide of potash to affect a case of tetanus, he would not hesitate to give a pound.

DR. HENRY C. DOUGHTY said that in those cases where cures had followed the excision of scars, it was because the center of infection was removed along with the cicatricial tissue. The reason that tetanus never followed railroad accidents when occurring along the line of road, but was often met with after injuries in the shops, was because the habitat of the tetanus bacillus was damp moist soil. It could not live in the sunlight, and hence was never present in the hard, dry, sun-baked earth of the road-bed.

DR. THOS. D. COLEMAN regarded woorara as the ideal agent for the control of tetanus owing to its action upon the peripheral nerves.

The President stated that the bacillus of tetanus could not live in the blood of living animals, and that the disease was a toxemia due to ptomaine absorption, and that quinine was one of the most powerful internal antiseptics known, exerting a most wonderful, neutralizing and antidotal effect upon all blood poisons; hence, its great curative power in tetanus, a fact pointed out many years ago by the late Dr. H. F. Campbell. As proving that tetanus neonatorum was a specific infectious disease, and not due to the rough handling of the naval, he cited the experiments of Escherich, who inoculated mice with a bit of tissue taken from near the umbilicus of an infant who had died of tetanus, and produced typical tetanic convulsions. He thought that the recent experiments of pathologists with antitetanin and other forms of attenuated virus offered a bright hope that this dread disease would, in the near future, be controlled and eradicated by scientific medicine.

